# Peroxiredoxins: hidden players in the antioxidant defence of human spermatozoa

**DOI:** 10.1186/2051-4190-24-4

**Published:** 2014-02-11

**Authors:** Cristian O’Flaherty

**Affiliations:** The Research Institute of the McGill University Health Centre, Montréal, Québec Canada; Department of Surgery (Urology Division), McGill University, Montréal, Québec Canada; Department of Obstetrics and Gynecology, McGill University, Montréal, Québec Canada; Department of Pharmacology and Therapeutics, McGill University, Montréal, Québec Canada; Urology Research Laboratory, Royal Victoria Hospital, room H6.46, 687 Avenue des Pins ouest, Montréal, Québec H3A 1A1 Canada

**Keywords:** Reactive oxygen species, Oxidative stress, Sperm function, Male infertility, Dérivés actifs de l’oxygène, Stress oxydant, Fonction des spermatozoïdes, Infécondité masculine

## Abstract

Spermatozoon is a cell with a precious message to deliver: the paternal DNA. Its motility machinery must be working perfectly and it should be able to acquire fertilizing ability in order to accomplish this mission. Infertility touches 1 in 6 couples worldwide and in half of the cases the causes can be traced to men. A variety of conditions such as infections of the male genital tract, varicocele, drugs, environmental factors, diseases, smoking, etc., are associated with male infertility and a common feature among them is the oxidative stress in semen that occurs when reactive oxygen species (ROS) are produced at high levels and/or when the antioxidant systems are decreased in the seminal plasma and/or spermatozoa. ROS-dependent damage targets proteins, lipids, and DNA, thus compromising sperm function and survival. Elevated ROS in spermatozoa are associated with DNA damage and decreased motility. Paradoxically, ROS, at very low levels, regulate sperm activation for fertilization. Therefore, the regulation of redox signaling in the male reproductive tract is essential for fertility. Peroxiredoxins (PRDXs) play a central role in redox signaling being both antioxidant enzymes and modulators of ROS action and are essential for pathological and physiological events. Recent studies from our lab emphasize the importance of PRDXs in the protection of spermatozoa as infertile men have significant low levels of PRDXs in semen and with little enzymatic activity available for ROS scavenging. The relationships between sperm DNA damage, motility and lipid peroxidation and high levels of thiol-oxidized PRDXs suggest the enhanced susceptibility of spermatozoa to oxidative stress and further support the importance of PRDXs in human sperm physiology. This review aims to characterize PRDXs, hidden players of the sperm antioxidant system and highlight the central role of PRDXs isoforms in the protection against oxidative stress to assure a proper function and DNA integrity of human spermatozoa.

## Résumé

Le spermatozoïde est une cellule qui porte un précieux message à délivrer : l’ADN paternel. La machinerie qui lui permet de se mouvoir doit fonctionner parfaitement et il doit avoir acquis ses capacités fécondantes afin d’accomplir sa mission. L’infertilité touche 1 couple sur 6 dans le monde, et dans la moitié des cas l’homme est en cause. Différentes conditions, telles que les infections du tractus génital masculin, la varicocele, les médicaments, les facteurs environnementaux, certaines maladies, la cigarette, etc., sont associées à l’infécondité masculine. Un trait commun à ces conditions est le stress oxydant dû sperme qui survient quand les dérivés actifs de l’oxygène (DAO) sont générés à des niveaux élevés et/ou quand les systèmes anti-oxydants sont diminués dans le plasma séminal et/ou dans les spermatozoïdes. Les dommages liés aux DAO touchent les protéines, les lipides, et l’ADN, ce qui compromet la fonction et la survie des spermatozoïdes. Des taux élevés de DAO dans les spermatozoïdes sont associés à une altération de l’ADN et à une diminution de leur mobilité. De façon paradoxale, les DAO à faibles concentrations contrôlent l’activation des spermatozoïdes lors de la fécondation. Ainsi la régulation de la signalisation redox dans le tractus génital masculin est essentielle pour la fécondité. Les peroxyrédoxines (PRDXs) jouent un rôle central dans la signalisation redox en étant à la fois des enzymes anti-oxydants et des modulateurs de l’action des DAO, se révélant ainsi essentielles aux processus pathologiques et physiologiques. Les travaux récents de notre laboratoire soulignent l’importance des PRDXs dans la protection des spermatozoïdes ; en effet, les hommes inféconds ont des taux significativement abaissés de PRDXs dans le sperme, avec une faible activité enzymatique disponible pour l’élimination des DAO. Les relations entre l’altération de l’ADN des spermatozoïdes, leur mobilité et la péroxydation des lipides, et les niveaux élevés de PRDX dont les résidus thiols sont oxydés suggèrent une forte susceptibilité des spermatozoïdes au stress oxydant et soulignent l’importance des PRDXs dans la physiologie des spermatozoïdes. Cette revue a pour objectifs de caractériser les PRDXs, acteurs cachés du système anti-oxydant des spermatozoïdes, et de mettre en lumière le rôle des isoformes des PRDXs dans la protection contre le stress oxydant pour assurer une fonction correcte et de préserver l’intégrité de l’ADN des spermatozoïdes humains.

## Introduction

Infertility is an important human health problem that affects ~15% of couples worldwide and the underlying cause in half of these cases can be traced to men [1]. Excessive levels in spermatozoa of reactive oxygen species (ROS) such as superoxide , hydrogen peroxide (H_2_O_2_), nitric oxide (NO^•^), the hydroxyl radical (HO^•^) and peroxynitrite (ONOO^-^), which are mostly produced in the sperm mitochondria [2] or by combination among them (NO^•^ and  produce ONOO-) and become injurious by-products of cellular metabolism [3–5], are associated with infertility [6–9]. Normally in somatic cells, elevated levels of ROS are prevented by the presence of a complex enzymatic antioxidant system involving superoxide dismutase (SOD) that removes  and catalase (CAT; restricted to peroxisomes), glutathione peroxidases (GPXs) and peroxiredoxins (PRDXs) that remove H_2_O_2_. GPXs and PRDXs are capable of removing peroxynitrite (formed by the combination of  and NO^•^). The oxidative stress, a condition resulting of an excessive production of ROS and/or a decrease in the antioxidant defense system [10, 11], may cause serious cell injury and even cell death [11, 12]. In the case of the spermatozoon, the oxidative stress targets all cell components decreasing sperm motility and mitochondrial activity [13, 14].

The infertile population has been increasing over the past few decades. However, treatment efficacy is poor because the underlying causes are unknown in 40-50% of cases [15]. Oxidative stress is a common feature of factors such as environmental pollutants, chemicals, drugs, smoke, toxins, radiation, and diseases related to infertility [16–19]. In such conditions, vital cell components, such as proteins, lipids, and DNA, are oxidized compromising cell function and survival [11, 12]. ROS-mediated damage to sperm is a significant contributing factor in 30-80% of infertile men [6–9, 20]. The antioxidant system present in semen [21, 22] is then not sufficient to protect sperm from ROS-dependent damage such as peroxidation of membrane lipids [23], DNA fragmentation and oxidation of bases [24, 25], low mitochondrial membrane potential [26, 27] and inactivation of enzymes associated with motility [28, 29].

In an era where the artificial reproductive techniques (ARTs), particularly the intracytoplasmic sperm injection (ICSI), are on rising, it is essential to use a safe sperm sample where the DNA integrity is not compromised. Significant sperm DNA oxidation is found in infertile patients and this type of damage has been associated with a wide range of reproductive outcomes from miscarriages to deliver of a live child [7, 30, 31]. It is worrisome the fact that a spermatozoon with significant DNA damage can be fertilize and even allow embryo development [31–33]. Therefore, it become a priority to perform more studies to gather information on the causes and consequences of oxidative DNA damage to avoid transmitting defects to the child through ARTs such as ICSI.

### ROS and male fertility

The transition from anaerobic to aerobic life came with a cost; the generation of ROS, active species that when produced at high amount promote cell dysfunction or, in extreme cases, cell death [11, 12]. However, ROS are beneficial molecules involved in cell signaling [34–37]. This is also true for spermatozoa; low levels of ROS are needed to accomplish capacitation, a process that the spermatozoon must undergo in order to achieve fertilizing ability [38–40]. During capacitation, ROS trigger and modulate protein phosphorylation events in a time dependent fashion [41, 42].

The presence of antioxidant enzymes is important to maintain low levels of ROS to avoid oxidative damage in spermatozoa [43–45]. Although it is evident that sperm function is regulated by redox signaling, how ROS production and action is modulated for sperm activation is still elusive. Semenogelin and zinc, present in high concentration in the seminal plasma, have been suggested as inhibitors of premature sperm capacitation [46, 47]. When capacitation takes place, these inhibitors are removed from the spermatozoa to allow a rise of ROS that will trigger the phosphorylation events that ultimate will allow the spermatozoon to achieve the capacitated state [48–50]. However, it is not known how the spermatozoon controls the levels of ROS to avoid the production of an excess of this reactive substances and thus promoting toxicity. Intracellular modulators of ROS production and action are currently unknown; however, a hidden family of antioxidant enzymes called peroxiredoxins (PRDXs) may play a fundamental role in the regulation of ROS action in spermatozoa.

### Peroxiredoxins, a new family of enzymes with more than antioxidant activity

**Figure 1:**
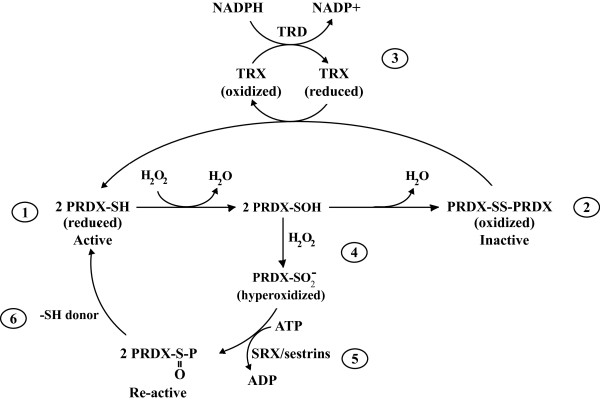
**Re-activation of PRDXs.** (1) PRDXs scavenge H_2_O_2_ and become oxidized and inactive (2). This inactivation is reversed by the thioredoxin (TRX)-TRX reductase (TRD) system that uses NADPH as reducing equivalents (3). (4) Further thiol oxidation of PRDXs by higher levels of H_2_O_2_ (hyperoxidation) radically inactivates the enzyme allowing H_2_O_2_ levels to increase in the cell and to trigger the H_2_O_2_-dependent signaling. This inactivation must be transient to avoid toxic effects by high levels of H_2_O_2_; thus, after transmission of the signal, SRX/sestrins re-activate PRDXs using ATP (P = phosphate group) (5). (6) Finally, donors of SH groups such as GSH or TRX, reduce PRDX.

PRDXs are ancestral SH-dependent, selenium- and heme-free peroxidases highly expressed in virtually all living species [51–53]. They are acidic proteins of ~20-31 kDa with one or two Cys residues at the active site, which are required for their activity [54]. In contrast to GPXs, PRDXs do not require metals ions for their activity [55–57]. They can reduce both organic and inorganic hydroperoxides [58], and peroxynitrite [59, 60] by coupling with the thioredoxins (TRX) TRX reductase (TRD) system [53, 61, 62] (Figure 1). PRDXs are direct targets for H_2_O_2_ due to their SH and thus are readily oxidized in cells exposed to low H_2_O_2_ levels [63–66]. PRDXs react with H_2_O_2_ as fast as GPXs [66, 67]; but PRDXs are known as the dominant peroxide-reducing enzymes in somatic cells [67–69].

PRDXs are regulators of redox signaling [35, 52, 70, 71]. The 2-Cys PRDXs are hyperoxidized to sulfinic acid and inactivated by H_2_O_2_ in diverse eukaryotes from yeast to mammals [35, 37, 52, 70, 72]. Hyperoxidized PRDXs are re-activated by the sulfinic acid reductases sulfiredoxin (SRX) and sestrins [72–75] (Figure 1). The present hypothesis states that once PRDXs are hyperoxidized, H_2_O_2_ concentration increases allowing the transmission of the signal [53, 62]. Then, PRDXs are re-activated by SRX and sestrins [72–76] and scavenge H_2_O_2_.

**Figure 2:**
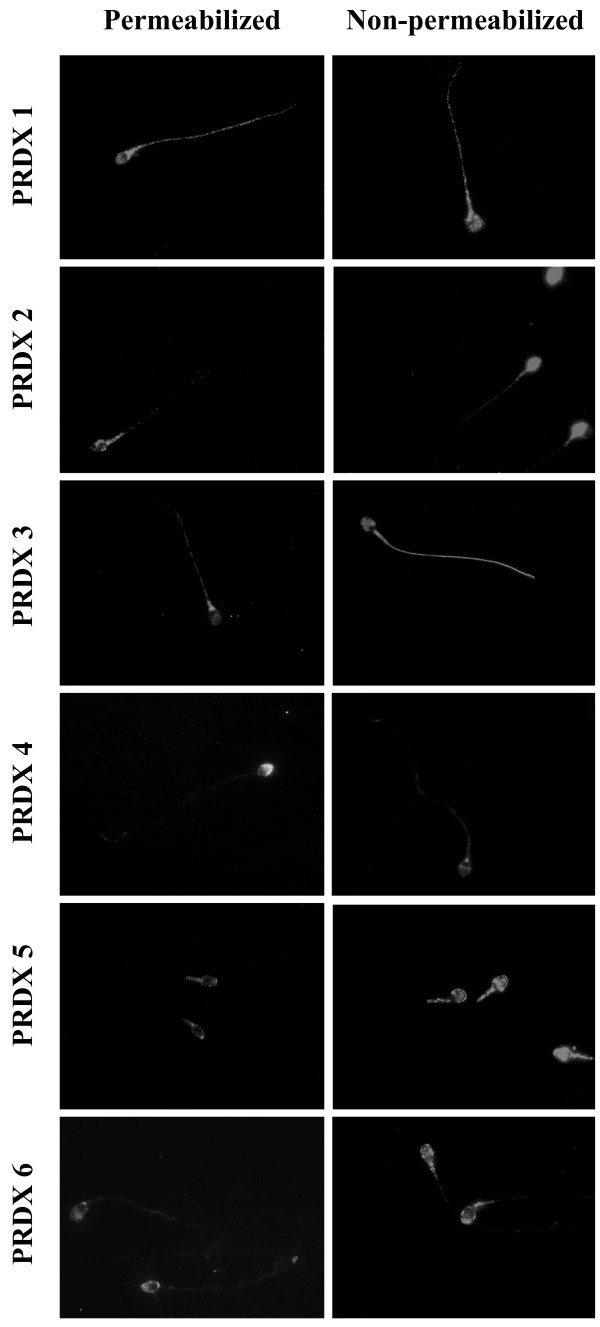
**Differential immunolocalization of PRDXs in human spermatozoa.** Spermatozoa were permeabilized or not with methanol and incubated overnight with the specific antibody against each PRDX and then with the corresponding biotin-labelled anti-mouse or anti-rabbit antibody followed by streptavidin conjugated to Alexa Fluor 555 [42]. The fluorescence obtained with the second antibodies and the Alexa Fluor 555-conjugated streptavidin alone was low and only at the level of the sperm head and equatorial segment (data not shown).

PRDXs are involved in processes such as cell cycle regulation, apoptosis, aging and cancer [77–79]. Animals lacking the PRDX1 gene are tumor prone [80], and their tissues contain elevated levels of damaged DNA [81]. Additionally, cellular senescence is accelerated in Prdx2^-/-^ mouse embryonic fibroblasts [82]. Spermatozoa from Prdx6^-/-^ mice are susceptible to oxidative stress [83]. PRDX4 is present in testis as two isoforms of 27 and 31 kDa [84]; the p27 form is found in the plasma membrane, cytosol and acrosome of human spermatozoa, whereas p31 is found in the head fraction, particularly in the acrosome (Figure 2, Table 1) [85]. The differences in solubility after treatment with detergents such as TritonX-100 (p27 isoform is soluble and p31 insoluble) [85] suggest differences in function; p31 is associated with the formation of the acrosome in the rat [84] and both isoforms are present in the perinucler theca of human spermatozoa [85]. Although the specific role of PRDX4 isoforms in these sperm structures is still unknown, it is possible to speculate that p27 may participate in the regulation of ROS levels and p31 function as a structural protein of the acrosome and perinuclear theca. Noteworthy, PRDX4 appears to have a protective role because mice lacking this isoform have testicular atrophy and increased sperm DNA damage [86].

**Table 1:** **Distribution of the known antioxidant enzymes in subcellular compartment of human spermatozoa**

***Plasma membrane***	***Cytosol***	***Acrosome***	***Nucleus***	***Equatorial segment***	***Midpiece***	***Flagellum***
PRDX2	PRDX4 (p27)	PRDX2	PRDX1	PRDX1	PRDX2	PRDX1
PRDX4 (p27)	PRDX6	PRDX4 (p27, p31)	PRDX2	PRDX5	PRDX3	PRDX2
PRDX5	Cu/ZnSOD	PRDX5	PRDX3	PRDX6	PRDX5	PRDX3
PRDX6	TRX1	PRDX6	PRDX4 (p27, p31)	TRX1	PRDX6	PRDX6
	TRD1	SPTRX1	PRDX5		MnSOD	SPTRX1
	TGR	SPRTX2	PRDX6		GPX4 (inactive)	SPTRX2
		TGR	GPX4 (inactive?)		TRX2	TRX-like 2
			SPRTX1		SPTRX1	TGR
			SPTRX2		SPTRX2	
			TRX1		TRD2	
			TGR		TGR	

References for enzymes listed above: PRDXs [85] and Figure 2, Cu/ZnSOD and MnSOD [87], GPX4 [88, 89], SPTRX1 [90–92], SPTRX2 [90, 91, 93], TRX2 and TRX-like-2 [91, 94], TRD1, TRD2 and TGR [90, 91].

The major role of PRDXs as H_2_O_2_ scavengers and sensors [10, 35, 37] is emphasized by their wide sub-cellular distribution (cytosol, nucleus, mitochondria, endoplasmic reticulum and plasma membrane [53, 62, 95–99]. Our results show that the same situation occurs in human spermatozoa (Figure 2 and Table 1) [85]. PRDXs are differentially distributed in all subcellular compartments of human spermatozoa; remarkably, at least two members of the family are present in each compartment (Table 1) [85]. This striking finding highlights the importance of PRDXs in sperm as major protectors against oxidative stress damage in spermatozoa and potentially key players on the regulation of the local action of ROS to sustain sperm function.

It is worth to mention that PRDX6 is highly abundant and the only member of the family present in all the subcellular compartments of human spermatozoa and to react with H_2_O_2_ at levels that promotes sperm capacitation [85], indicating that PRDX6 might be major player in the regulation of sperm activation.

Traditionally, it is considered that CAT and GPXs are the major if not the unique antioxidant enzymes to protect spermatozoa. This statement was supported by measuring the enzymatic activity by using either H_2_O_2_ (for CAT) or organic hydroperoxides in a reaction with GSH and coupled with glutathione reductase/NAPDPH system to re-cycle the GSSG to GSH (for GPXs). Then, the decay of absorbance due to H_2_O_2_ or NADPH consumption is considered as a measurement of CAT or GPX activities, respectively. Because active PRDXs are present in human spermatozoa [43, 85], caution must be taken when these assays are use to determine antioxidant enzymatic activities in these cells. PRDXs can account for the enzymatic activity obtained using these assays as they can use H_2_O_2_ or organic hydroperoxides, NADPH and GSH for their activity. The use of inhibitors such as carmustine (inhibitor of glutathione reductase (GRD)) and diethylmaleate (binds to GSH making it non-accessible for GPX/GRD system) are useful to determined specifically PRDX activity [100].

**Figure 3:**
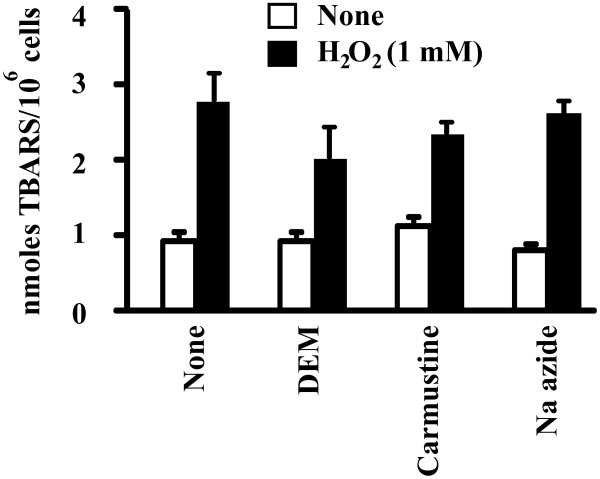
**Lipid peroxidation is not increased in human spermatozoa treated with 5 mM diethylmaleate (DEM; binds to GSH making it non-accessible for the GPX-GRD system), 50 μM carmustine (inactivates glutathione reductase and diaphorase activity) or 50 μM NaN**_**3**_ **(inhibitor of catalase).** Lipid peroxidation was measured by spectrofluorometry according to Aitken et al. (1993) [103]. Spermatozoa from 4 different healthy donors were used in this experiment. The presence of none of the inhibitors used increased the level of lipid peroxidation in human spermatozoa (results were analyzed by ANOVA; p < 0.05).

Based on our findings and on what was explained above, it can be concluded that PRDXs are the first line of defence against H_2_O_2_ and other ROS (hydroperoxides, peroxynitrite) for human spermatozoa because their H_2_O_2_ scavenging capacity [101] (Table 2) does not seem to involve CAT (peroxisomes that contain the enzyme are eliminated from germ cells during spermatogenesis [102], and sodium azide (catalase inhibitor) did not reduce that H_2_O_2_ scavenging capacity (Table 2) or increased the level of sperm lipid peroxidation (Figure 3)).

**Table 2:** **Sperm H**_**2**_ **O**_**2**_ **scavenging capacity is not prevented by sodium azide, inhibitor of catalase**

	Units/10^8^ spermatozoa
Sperm extract	2.2 ± 0.4
Sperm extract + 50 μM NaN_3_	2.6 ± 0.3

One unit of H_2_O_2_ scavenging capacity is defined as the quantity of spermatozoa capable of decreasing the amount of H_2_O_2_ present in solution by 50%. Results obtained from 3 healthy donors.

Glutathione peroxidase 2, 3 and 5 are not found in human spermatozoa, testes or seminal plasma [104, 105] and GPX4 is part of the mitochondrial sheath and it is enzymatically inactive in mature spermatozoa [88, 89, 106]. The role of GPX1 in human sperm is controversial because GPX1 activity was measured using cumene hydroperoxide and NADPH [107], substrates also used by PRDXs. In any case, the participation of GPXs in the antioxidant protection of human spermatozoa might be limited since treatment with diethylmaleate or with carmustine, do not increase the level of lipid peroxidation (Figure 3). Therefore, at least for human spermatozoa, oxidative stress and the associated damage is handled by PRDXs isoforms.

### Peroxiredoxins and male infertility

Human spermatozoa are highly sensitive to ROS [9, 14, 108]. This particularity is due to high content of polyunsaturated fatty acids in the plasma membrane, target for extensive oxidation, little cytoplasm and thus low capacity for antioxidant protection by cytoplasmic enzymes (e.g. Cu-Zn SOD) and limited DNA repair mechanisms [22, 109–111]. These deficiencies can be worsen if the PRDX system is altered by oxidative stress; because PRDXs are easily oxidized even at low ROS concentration, the presence of an oxidative stress in human spermatozoa will alter the capacity of these enzymes to scavenge excessive amounts of ROS. The necessity for active PRDXs is supported by the data from infertile men that have significant lower amounts of PRDXs in both seminal plasma and spermatozoa compared to healthy donors [43]. PRDX6, but not PRDX1, is present in low amounts in seminal plasma of infertile men with clinical varicocele. The total quantity of PRDX1 and PRDX6, but not for PRDX4 and PRDX5, is lower in spermatozoa from varicocele patients (prior to surgery) than in idiopathic infertile men or healthy donors [43]. In terms of PRDXs expression in spermatozoa, the population of infertile men is heterogeneous; sperm PRDX6 was low in 67% and 39% varicocele and idiopathic infertile patients, respectively, whereas sperm PRDX1 was only low in 42% of varicocele patients [43]. Noteworthy, thiol-oxidized PRDX1, PRDX5 and PRDX6 levels were higher in spermatozoa from idiopathic infertile men than from donors [43]. Due to the lower amount of total PRDX1 and PRDX6 and the high thiol oxidation of these PRDXs, very little (less than 20%) protection due to PRDXs remains and this is associated with impaired sperm motility and poor DNA quality [43].

**Figure 4:**
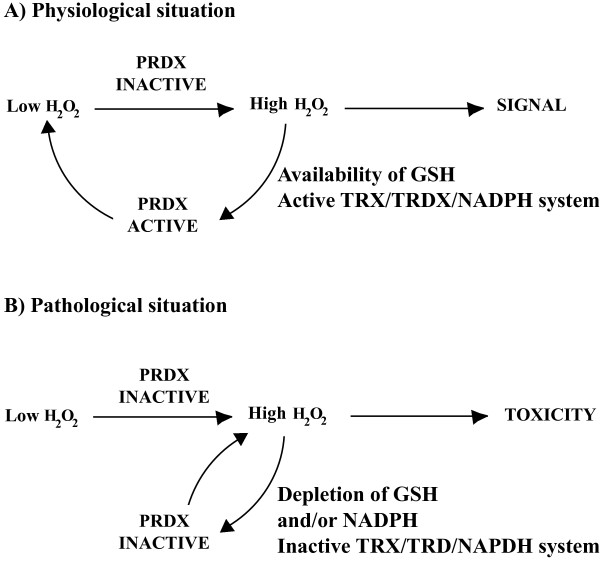
**Consequences of PRDXs inactivation in physiological and pathological situations. A)** The inactivation of PRDXs due to an increase of H_2_O_2_ occurs in order to allow the signaling for physiological processes. After the signal is triggered, the GSH and NADPH availability and the presence of an active TRX/TRD system allow the re-activation of PRDX to decrease the intracellular level of H_2_O_2_. **B)** Under pathological situations, the already high H_2_O_2_ levels increase even more as the GSH and NAPDH are depleted. Therefore, PRDXs remain inactive and in consequence, the spermatozoon is not protected against the H_2_O_2_-dependent damage on lipids, proteins and DNA.

Interestingly, sperm levels of high molecular mass complexes of hyperoxidized PRDX6 were higher in both infertile men groups than in donors and the PRDX6 thiol oxidation ratio correlated with levels of lipid peroxidation in spermatozoa [43]. From these studies it is evident that thiol oxidation of PRDXs is associated with impairment of sperm function. It is possible that, due to an inability to reduce PRDXs, the levels of ROS rise at toxic levels in the spermatozoon and thus promoting infertility (Figure 4). The potential scenario of failing re-activation of PRDXs is very much plausible in the spermatozoon; the availability of glutathione is minimal in this cell since the level of GSH in human spermatozoa is ~0.3 mM compared to the 10 mM concentration that can be found in somatic cells [3, 112]. Since GSH is necessary to reduced PRDX6 [113], due to this limitation the reduction of PRDX6 is jeopardized if there is a strong oxidative stress in the spermatozoon and GSH is depleted. The 2-Cys PRDXs can be re-activated by the TRX/TRD system; however, this system is limited by the amount of NADPH present in the cell. In cases of oxidative stress, glucose 6-phosphate dehydrogenase, generator of NADPH, is inactivated and thus the amount of NADPH is rapidly depleted and in consequence this reducing equivalent is no longer available [114]. Recently, it was reported that aging mice lacking SPTRX1 and SPTRX2 are subfertile [115]. This findings support the need for an intact TRX system to assure fertility. In human spermatozoa, SPTRX1 and SPTRX2 as well as TRX like 2 have been described and their localization is summarized in Table 1. The presence of TRD1 and TRD2 (enzymes that reduce the oxidized TRXs including SPTRXs) has been demonstrated by immunoblotting [90], but the exact localization in the sperm cell is still unknown. These studies confirm that a TRX/TRD system is in place in human spermatozoa and more research must be done to better characterize the interaction of PRDXs with this system in the different subcellular compartments of the ejaculated spermatozoon. Another thioredoxins reductase called thioredoxin glutathione reductase (TGR) has been found in human spermatozoa and may be also contributing to the reduction of TRXs [91].The hyperoxidized 2Cys PRDXs can be re-activated by SRX or sestrin with energy consumption [72–75]. Immunohistochemistry studies revealed a moderate staining for SRX (in Sertoli, Leydig and germ cells) and strong staining (mostly in Sertoli and Leydig cells) and moderate for germ cells for sestrin-2 [116]; however, the presence of these enzymes is yet to be confirmed in mature spermatozoa.

Based on what was discussed above, the data presented stress the importance of PRDXs in antioxidant protection of the spermatozoon and offer a possible cause for impaired sperm function in infertile patients. Moreover, the different amounts of PRDXs and their thiol oxidation status in spermatozoa among infertile men may serve the basis for the development of new diagnostic tools.

### Antioxidant therapy

Knowing that the oxidative stress plays a major role in the pathophysiology of male infertility [7, 20, 117], the antioxidant therapy seems to be the logical strategy to treat those patients. Limited controlled studies, with low number of subjects support the use of antioxidants to treat infertility [118, 119]. Although in some causes fertility has been improved, in some cases the use of these compounds may harm rather than to help the spermatozoon; for instance the administration of a combination of antioxidants to infertile patients promote a decrease in the DNA compaction and thus exposing the DNA to further damage [120]. There is no doubt that the administration of antioxidant may help infertile patients to achieve fatherhood, but it is essential to know more about the redox signaling is regulated in human spermatozoa to avoid the interference of these compounds on sperm physiology. It is needed a deeper study of what ROS and their levels, and what antioxidant enzymes are impaired in cases of male infertility to design a more ‘directed’ or ‘customized’ antioxidant therapy. The use of ‘antioxidant cocktails’ that is not always beneficial for infertile patients support this re-thinking on how ROS must be controlled in infertile patients.

## Conclusions

The human spermatozoon is extremely sensitive to high levels of ROS. In order to keep in line these active molecules, it should be a very tuned PRDX and TRX/TRD systems working together in regulating the levels of ROS to avoid impairment of sperm function. Their wide distribution in every compartment of the sperm and the association of low levels and inactivation of them and low sperm quality makes PRDXs a major players in the antioxidant protection and the modulation of ROS action in human spermatozoa.

## Authors’ information

CO is assistant professor at the Department of Surgery, Urology Division, McGill University and medical scientist at the Research Institute, McGill University Health Centre.

## Abbreviations

ATP: Adenosine triphosphate
Cys: Cysteine residue
DEM: Diethylmaleate
DTT: Dithiothreitol
GPX: Glutathione peroxidase
GRD: Glutathione reductase
GSH: Glutathione, reduced form
H_2_O_2_: Hydrogen peroxide
MnSOD: Manganese superoxide dismutase (SOD2)
NADP+: Nicotinamide adenine dinucleotide phosphate, oxidized form
NADPH: Nicotinamide adenine dinucleotide phosphate, reduced form
NaN_3_: Sodium azide
O_2_^•-^: Superoxide anion
ONOO: Peroxynitrite
PRDX: Peroxiredoxin
ROS: Reactive oxygen species
SH: Sulfhydryl (thiol) group
SO2: Sulfinic acid group
Cu/ZnSOD: Cooper/zing superoxide dismutase (SOD1)
SRX: Sulfiredoxin
SS: Disulfide
TBARS: Tiobarbituric acid reactive substances
TRD: Thioredoxin reductase
TRX: Thioredoxin
TGR: Thioredoxin glutathione reductase
SPTRX: Sperm specific thioredoxin.
